# The 2025 United States Measles Crisis: When Vaccine Hesitancy Meets Reality

**DOI:** 10.7759/cureus.88196

**Published:** 2025-07-17

**Authors:** Adekunle F Adeoye, Daniel O Umoru, Ousainou O Gomez, Isreal A Onifade, Benjamin O Akangbe, Ubalaeze S Elechi, Vincent U Barrah

**Affiliations:** 1 Public Health, Georgia State University, Atlanta, USA; 2 Pharmacy, National Hospital Abuja, Abuja, NGA; 3 Biostatistics and Epidemiology, Georgia State University, Atlanta, USA; 4 Biology, University of Albany, Albany, USA; 5 Radiology, Lifechart Medical Diagnostic Services, Enugu, NGA; 6 Digital Health, University of Nigeria, Enugu, NGA; 7 Public Health, Chicago State University, Chicago, USA

**Keywords:** measles, public health coverage, united states of america, vaccination coverage, vaccine hesitancy

## Abstract

This narrative review examines how declining vaccine uptake and growing vaccine hesitancy created pockets of susceptibility that enabled the outbreak and analyzes the epidemiological features and public health response. Key drivers of vaccine hesitancy (misinformation about vaccine safety, philosophical and religious exemptions, and pandemic-related disruptions) are discussed, along with the demographic patterns of under-vaccination in the U.S. Comparisons are drawn to notable measles outbreaks in Samoa (2019) and the United Kingdom (2018) to highlight common themes of insufficient immunity leading to severe outcomes. The review evaluates what aspects of the public health response were effective, including emergency vaccination campaigns, community engagement, and interagency coordination, and what challenges and failures impeded control, such as resource limitations and delayed counter-misinformation efforts. The economic impact of the outbreak is also considered, with containment efforts costing millions of dollars. We summarize the lessons learned about the importance of maintaining high vaccination coverage and suggest policy implications, which include stricter immunization requirements, proactive education to combat misinformation, and strengthening immunization infrastructure. Finally, future directions and recommendations are presented to prevent measles from regaining a foothold, emphasizing that sustained commitment to vaccination and public trust is critical to averting similar crises.

## Introduction and background

Measles, once a ubiquitous childhood infection in the U.S., was declared eliminated nationally in 2000 after decades of widespread vaccination and vigilant surveillance [1]. Yet elimination never guaranteed permanence. During the 2010s, an erosion of public confidence in vaccines, amplified by online misinformation, chipped away at the herd immunity that had suppressed endemic transmission [2]. Concerningly, kindergarten coverage with the recommended two doses of the measles-mumps-rubella (MMR) vaccine fell from 95.2% in the 2019-2020 school year to 92.7% in 2023-2024, leaving an estimated 280,000 children susceptible [3].

The consequences of those immunity gaps became starkly apparent in early 2025. On 30 May 2025, the Centers for Disease Control and Prevention (CDC) reported 1,088 confirmed measles cases across 33 jurisdictions, including the first measles-related deaths recorded in the country since 2015 [4]. More than 90% of patients were unvaccinated or had unknown vaccination status, and nearly all cases were epidemiologically linked to undervaccinated communities in Texas, New Mexico, and Oklahoma. Air travel and domestic tourism quickly distributed the virus to additional states, illustrating how a single imported case can ignite nationwide spread when local protection is uneven.

Although the absolute numbers remain modest compared with the pre-vaccine era, the 2025 outbreak represents the largest U.S. measles crisis in a decade and a critical stress test of the nation’s vaccine policy architecture. It also mirrors recent international setbacks, notably the 2018 United Kingdom resurgence and the 2019 Samoa epidemic, underscoring that measles control is precarious wherever vaccination falters.

In this narrative review, we synthesize current evidence to elucidate (1) the historical and epidemiologic context of the 2025 U.S. outbreak, (2) the socio-behavioral and policy drivers of vaccine hesitancy that primed the crisis, (3) the strengths and weaknesses of the public health response, (4) the human and economic costs incurred, and (5) the lessons and forward-looking strategies required to restore and sustain measles elimination. By integrating national data with insights from global comparators, we aim to furnish clinicians, policymakers, and public health practitioners with a comprehensive blueprint for preventing measles from regaining a foothold in the U.S.

## Review

Methods

For this narrative review, we conducted a comprehensive literature search of PubMed, Google Scholar, and official health organization websites (CDC, WHO) to identify sources on measles outbreaks, vaccination coverage, vaccine hesitancy, and public health responses published between 2010 and 2025. Sources were eligible if they (i) contained primary or secondary data on measles epidemiology, vaccination coverage, or hesitancy; (ii) were published in English during 2010-2025; and (iii) offered a verifiable DOI or permanent URL. Editorials without data, conference abstracts, and duplicate preprints were excluded. Search terms included combinations of “measles,” “outbreak,” “vaccine hesitancy,” “measles 2025,” “vaccination coverage,” “measles mortality,” and “measles elimination.” We prioritized peer-reviewed articles (epidemiological studies, reviews, commentaries) and authoritative reports (e.g., Morbidity and Mortality Weekly Report (MMWR) briefs, WHO updates), with particular focus on data from the 2025 U.S. outbreak and comparator events such as the 2019 Samoa epidemic and the 2018 measles resurgence in Europe. Outbreak case counts and vaccination-coverage figures were cross-checked against the CDC and WHO databases for accuracy. All U.S. 2025 figures reflect data current as of 30 May 2025.

Historical perspective and outbreak epidemiology

Measles elimination in the U.S. was achieved in 2000 through decades of high vaccine coverage and aggressive public health campaigns [5]. In the years immediately after, only a handful of cases occurred annually, typically in travelers or their close contacts. However, by the 2010s, declining vaccination in certain communities led to a series of outbreaks. For example, in 2017, a measles outbreak in Minnesota’s Somali-American community, where unfounded fears about MMR causing autism had spread, resulted in 75 cases [6]. These incidents were a prelude to a larger resurgence in 2019, when the U.S. recorded 1,282 cases, the most since 1992 [7]. That year, the vast majority of cases were confined to under-immunized enclaves, including a prolonged outbreak of 934 cases in Orthodox Jewish neighborhoods in New York that nearly cost the country its elimination status. Although no measles deaths occurred in 2019, the outbreaks were a wake-up call that highlighted growing vaccine complacency.

This American pattern mirrored global trends. Europe saw measles cases surge in 2018-2019, and the United Kingdom lost its measles-free status after a protracted outbreak (991 cases in 2018) reestablished endemic transmission [8]. In late 2019, Samoa experienced a devastating measles epidemic, with over 5,700 cases and 83 deaths, after national MMR coverage fell to around 30% [9]. These examples demonstrate that if vaccination rates decline, measles can resurface and cause significant harm.

Outbreak epidemiology

**Figure 1:**
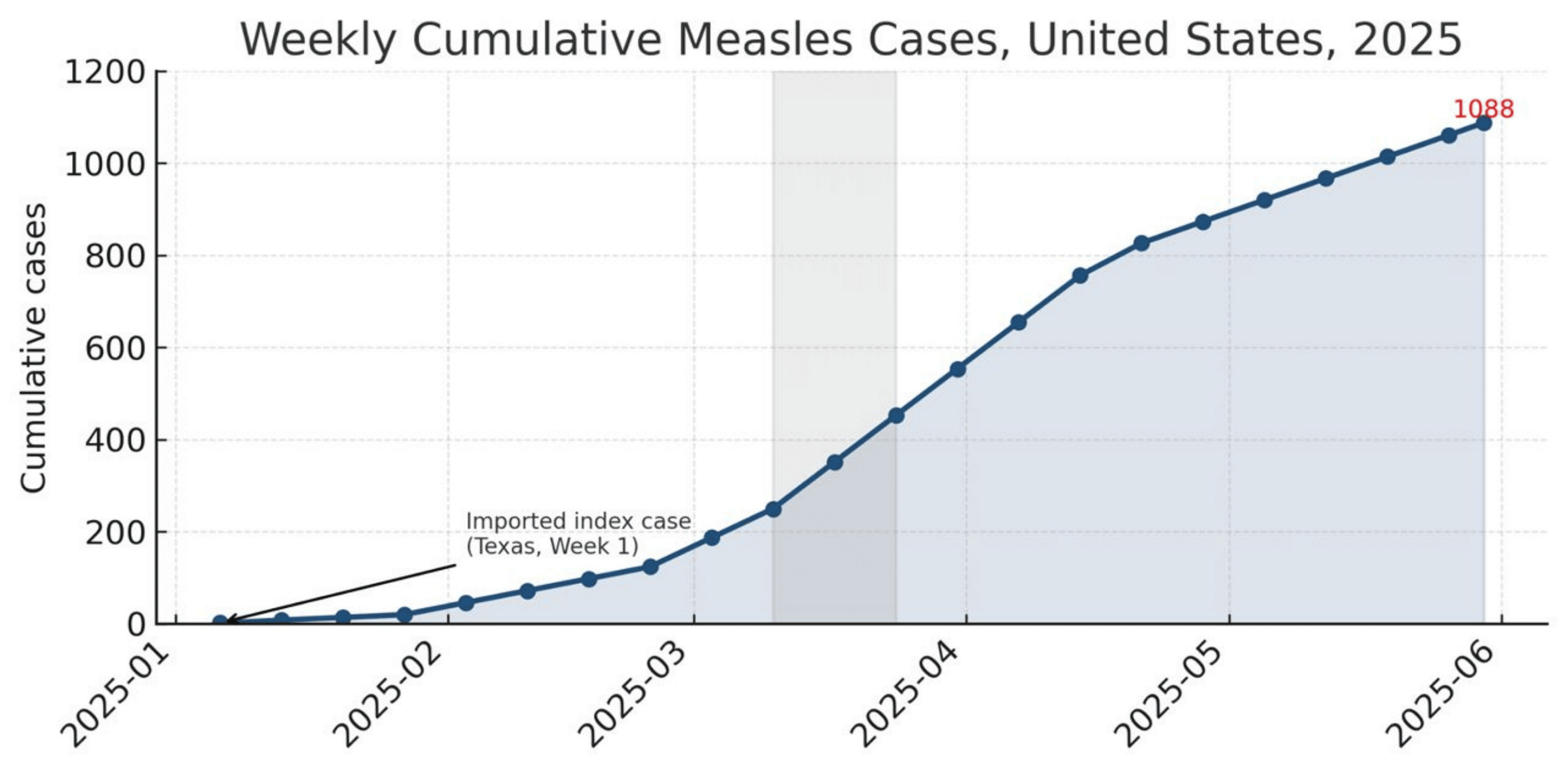
Weekly Cumulative Measles Cases, United States, 2025 Weekly cumulative measles cases in the United States, 1 January-30 May 2025. The outbreak begins with an imported index case in Texas during the first week of January, accelerates sharply through March, peaks in mid-April, and slows after nationwide response measures were intensified in mid-March (gray band). Image credit: Ubalaeze Solomon Elechi, created with OpenAI’s GPT-4 and DALL-E tools

The 2025 U.S. measles outbreak began in early January, seeded by an infected traveler in a close-knit, undervaccinated community in West Texas. From there, the virus spread across at least three states (Texas, New Mexico, and Oklahoma) among populations with historically low MMR uptake. By 17 April 2025, the CDC had reported 800 cases nationally, already the second-highest annual total in 25 years, and it was noted that 82% were linked to the outbreak in those three states [10]. Despite control efforts, the case count continued to rise; as of 29 May 2025, a total of 1,088 measles cases had been confirmed across 33 states [11]. Fourteen distinct outbreaks (defined as three or more linked cases) were identified in 2025, though the majority of all cases were associated with the large Texas-centered outbreak. Figure 1 depicts the weekly accumulation of cases from 6 January through 30 May 2025, illustrating the rapid March-to-April growth phase and subsequent deceleration.

**Figure 2:**
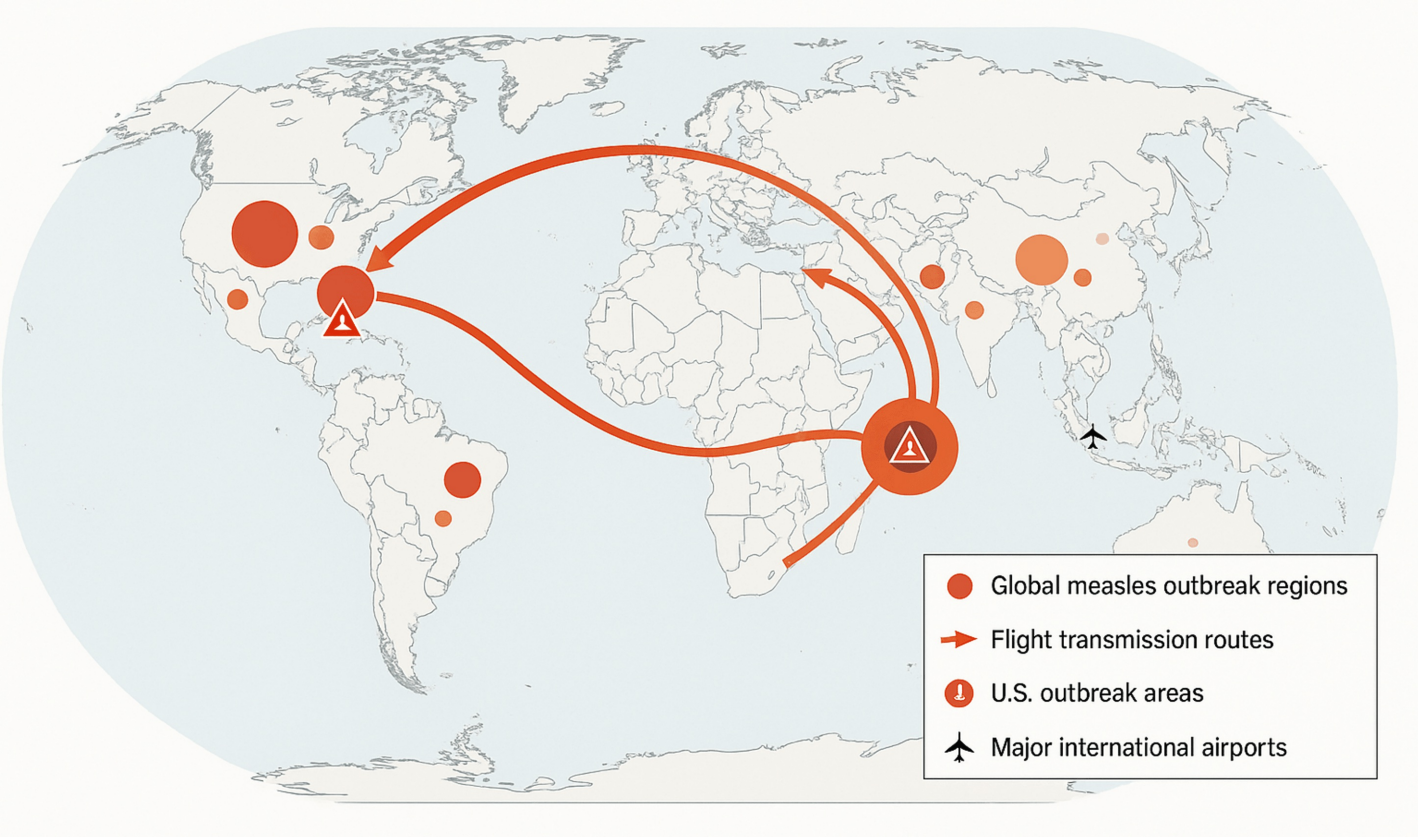
International Measles Transmission Pattern Conceptual illustration of measles spread through international air travel. In 2025, imported measles cases (infected travelers arriving in the U.S.) ignited outbreaks in undervaccinated communities. Global travel has linked measles outbreaks across continents, underscoring that high vaccination coverage is needed everywhere to prevent cross-border spread. Image credit: Ubalaeze Solomon Elechi, created with OpenAI’s GPT-4 and DALL-E tools

The epidemiological profile of the outbreak highlights the consequences of immunity gaps. More than 90% of patients were unvaccinated or had unknown vaccination status (per CDC reports), and many belonged to communities that had resisted routine immunizations. Cases spanned all age groups, but young children were heavily affected: roughly one-third of cases were in children under five (including many infants too young to be vaccinated), another third in school-aged children, and the remainder in adults. About 1 in 10 patients required hospitalization for measles complications or isolation. Most tragically, three measles-related deaths occurred (two children and one immunocompromised adult), the first U.S. measles fatalities in over 20 years [12]. Genomic analyses confirmed that the outbreaks stemmed from imported virus strains, not a new endemic variant [13]. Notably, travel played a role in amplifying the spread: for instance, several cases in Colorado and Illinois were traced to exposures during domestic airline flights and at tourist destinations [14]. These scenarios illustrated how quickly measles can hop between susceptible pockets. As illustrated in Figure 2, sustained measles activity in 2025 global hotspots, particularly parts of Europe, Southeast Asia, and sub-Saharan Africa, generated multiple air-travel corridors into major U.S. airports, ultimately funneling cases into the undervaccinated Texas-New Mexico-Oklahoma cluster that seeded the domestic outbreak. Conversely, areas with high vaccination coverage largely escaped secondary transmission, demonstrating the protective value of herd immunity where it remained intact.

Comparative Analysis: Affected Versus Unaffected States

As of 30 May 2025, the three core outbreak states (Texas, New Mexico, and Oklahoma) recorded a mean incidence of 7.4 cases per million residents, whereas the remaining 47 jurisdictions averaged 0.06 cases per million, a 120-fold difference [11]. States in the lowest kindergarten MMR-coverage quartile (≤ 92%) accounted for 87% of all U.S. cases, while no state with ≥ 96% coverage reported more than five cases [3]. This inverse relationship between coverage and incidence mirrors modeling work showing that every percentage-point drop in two-dose coverage increases outbreak probability by roughly 14% [15].

Granular Demographic Breakdown

The U.S. 2025 cases had a demographic profile of 1,088 individuals. Among line-listed patients, 34% were children under five years of age, 33% were school-aged (5-17 years), and 28% were adults ≥ 18 years; the remaining 5% had age unreported [11]. Hospitalization was required for 11% of cases overall, rising to 18% in infants < 12 months. Vaccination status was known for 1,012 patients: 86% were unvaccinated, 7% had received only one MMR dose, and 7% were fully vaccinated, yielding an estimated two-dose effectiveness of 97% against confirmed disease. Sex distribution was balanced (51% male). Racial/ethnic data, available for 62% of cases, showed over-representation of Hispanic/Latino children in the Texas and New Mexico clusters, while non-Hispanic White children predominated in the Oklahoma and Colorado clusters [11].

Vaccine hesitancy: drivers and demographics

Measles outbreaks in the 21st century have been overwhelmingly linked to clusters of unvaccinated individuals, raising the question: Why are people forgoing a readily available, life-saving vaccine? The drivers of vaccine hesitancy are multifaceted. Foremost is misinformation about vaccine safety, notably the debunked claim linking the MMR vaccine to autism, originally propagated by a fraudulent study in 1998 [16]. Fear of autism remains a leading reason cited by parents who refuse MMR for their children. Other parental concerns include perceived risks of vaccine side effects, the belief that “natural” immunity is healthier, and general distrust of pharmaceutical companies or government health agencies. Social media and online echo chambers have amplified vaccine misinformation, allowing anti-vaccine activists to sow doubt about the MMR vaccine’s safety despite an overwhelming scientific consensus of its safety and efficacy. The result has been a measurable decline in public confidence in childhood vaccines in some communities. The WHO identified vaccine hesitancy as one of the top 10 threats to global health in 2019 [2], acknowledging that complacency and misinformation were undermining decades of progress. In the U.S., the COVID-19 pandemic may have further fueled hesitancy; not only did routine pediatric vaccination rates dip during pandemic lockdowns, but polarized debates around COVID-19 vaccines spilled over to routine immunizations, reinforcing doubts among some groups [17].

Legacy of the MMR-Autism Myth

Persistent concern that the MMR vaccine causes autism remains the single most frequently cited reason for MMR refusal in U.S. parent surveys [18]. Evidence refuting this link is unequivocal: a 2014 population-based meta-analysis of over 1.2 million children found no association between MMR exposure and autism spectrum disorder (ASD) [19]. A nationwide Danish cohort study covering 657,461 births likewise reported a null association (adjusted hazard ratio = 0.93; 95% confidence interval (CI): 0.85-1.02) [20]. Most recently, an updated U.K. matched-cohort analysis reaffirmed that ASD incidence is identical in vaccinated and unvaccinated children [21]. Despite this consensus, online recirculation of the discredited 1998 Wakefield paper continues to seed hesitancy within some communities, indicating the importance of proactive, evidence-based communication strategies.

Demographics

Hesitancy is not uniform across the population; it clusters in specific demographics and communities. Paradoxically, areas with higher socioeconomic status and education levels have at times harbored more vaccine-refusing parents. For example, communities in California and Colorado, where “natural lifestyle” movements flourish, saw MMR vaccination rates fall well below 90% before policy changes. In the 2023-2024 school year, the national average MMR coverage for kindergarteners was 92.7%, but this average masks geographic pockets of much lower coverage [3]. Fourteen U.S. states reported that over 5% of their kindergarten students had non-medical vaccine exemptions in 2023-2024, with the national exemption rate rising to 3.3% [22]. These exemptions, often due to personal or religious belief waivers, tend to concentrate in certain schools and communities, creating pockets of susceptibility. Religious or cultural beliefs also play a role: insular religious communities (such as the Amish, some Orthodox Jewish groups, and certain fundamentalist churches) have experienced measles outbreaks after members declined vaccines for faith-related reasons [23]. Minority communities can face unique challenges; for instance, Somali-American families in Minnesota were targeted by anti-vaccine misinformation about autism, leading to a dramatic drop in MMR uptake and a subsequent measles outbreak in 2017 within that community [23]. Additionally, structural barriers and healthcare access inequalities mean some children miss vaccinations not by choice but due to healthcare system gaps, although the Vaccines for Children program and public health clinics aim to minimize this in the U.S.

The demographics of the 2025 measles outbreak reflect these hesitancy patterns. Several West Texas counties at the center of the 2025 outbreak have carried kindergarten two-dose MMR coverage well below the 90% threshold for multiple consecutive years: Briscoe County, TX, 80.0%; Childress County, TX, 70.5%; and Dawson County, TX, 88.1%, compared with the 2023-2024 national average of 92.7% [24,25]. CDC line-list data show that through 17 April 2025, 69% of U.S. measles cases occurred in persons < 20 years of age (38% aged 5-19 years and 31% aged < 5 years), and over 90% of all patients were either unvaccinated or of unknown vaccination status [10,11], patterns consistent with pockets of routine-immunization refusal in the affected communities. Meanwhile, several adult measles cases in the 2025 outbreak were individuals who had never been vaccinated due to similar beliefs or who missed childhood vaccination and lacked awareness of the need. Ultimately, the outbreak was driven by immunity gaps rather than any change in the virus or the vaccine. Vaccine hesitancy, fueled by fear, misinformation, and sociocultural dynamics, supplied the tinder, and a single imported case ignited an epidemic in these unprotected communities.

Public health response and economic impact: what worked, what failed

Containing the 2025 outbreaks required a swift and coordinated public health response. Local, state, and federal agencies implemented classic measures: prompt isolation of cases, contact tracing, and targeted vaccination of susceptible contacts and communities [26]. Health departments in Texas and neighboring states held emergency immunization clinics and, in affected counties, even recommended an early MMR dose for infants 6-11 months old [27]. Schools in outbreak areas excluded unvaccinated students during the incubation period, and public health officials issued travel advisories and alerts to clinicians. These efforts, alongside extensive community outreach, eventually curbed the spread by early summer 2025 [11]. As summarized in Table 1, the 2025 U.S. outbreak’s scale was comparable to recent high-profile resurgences in Samoa (2019) and the United Kingdom (2018), underscoring how gaps in coverage translate into costly public-health responses.

**Table 1:** Key Measles Outbreaks in Recent Years (Selected Examples) Note: Immunization coverage refers to the estimated national or subnational uptake of the first and/or second dose of MMR vaccine prior to each outbreak. Data were compiled from the CDC and WHO reports. "Pockets of low coverage" denote geographic or demographic subgroups with significantly lower-than-average vaccine uptake, which contributed to outbreak vulnerability. MMR: measles, mumps, and rubella vaccine; WHO: World Health Organization; U.S.: United States

Location (Year)	Cases	Deaths	Immunization Coverage (Pre-outbreak)	Notable Characteristics
United States (2019)	1,282	0	~94% nationwide; pockets of low coverage (some communities < 80%)	Largest U.S. measles outbreak since 1992; concentrated in under-vaccinated Orthodox Jewish communities (~75% of cases); elimination status narrowly preserved [7].
United States (2025)	1,088	3	92.7% nationwide; local pockets < 70%	Worst U.S. outbreak since 2019 (ongoing); ~90% of cases in unvaccinated persons; includes a major outbreak in West Texas/New Mexico (82% of 2025 cases) [11].
United Kingdom (2018)	991	3	~95% (first dose), ~87% (second dose) at school entry	Lost WHO “measles-free” status after a prolonged outbreak reestablished endemic transmission; measles cases doubled from 2017 to 2018, with several fatalities in unvaccinated children [8].
Samoa (2019)	5,707	83	~30% (estimated MMR coverage before outbreak)	Nationwide epidemic following a dramatic drop in vaccination; > 80% of deaths in children < 5 years; state of emergency declared with mass vaccination campaign to raise coverage [9].

Certain response strategies clearly worked. Rapid case identification through vigilant surveillance and laboratory testing allowed timely interventions. Interagency communication (fortified by pandemic-era coordination frameworks) facilitated resource sharing, and community leaders were engaged to promote vaccination in hesitant populations [26]. The fact that measles did not become endemic beyond the initial clusters is a testament to these control measures. However, the outbreak also exposed gaps. Years of inadequate investment in vaccine education meant that by the time officials mobilized messaging in 2025, misinformation was deeply entrenched in the hardest-hit communities [28]. Understaffed local health departments struggled with the surge in contact tracing and immunization workloads, reflecting a broader disinvestment in public health infrastructure. Jurisdictional complexities (for example, coordinating quarantine across state lines) at times hampered the speed of response. On 7 March 2025, the CDC issued Health Alert Network Advisory #00522 instructing jurisdictions to isolate suspected cases, trace contacts, and administer post-exposure MMR within 72 hours or immune globulin within six days [26,29]. Within a week, the Texas Department of State Health Services (DSHS) circulated the same protocol to county health departments [30]. Yet rural West Texas counties reported that vaccine stockpiles were depleted and that only one or two immunization nurses were available for surge duty, producing contact-tracing backlogs of 48-60 hours [31,32]. The 2022 NACCHO Profile Survey had already found that 51% of U.S. local health departments lacked sufficient staff to manage vaccine-preventable disease (VPD) surges [33], a gap echoed in Texas after-action reviews. Meanwhile, the CDC’s advisory recommended furlough of exposed but non-immune healthcare workers; several rural hospitals complied yet could not backfill shifts, forcing diversion of febrile-rash patients to distant facilities [34]. These examples illustrate how robust federal guidance can be blunted when county-level workforce and vaccine reserves are inadequate [28].

The economic impact of the outbreak was substantial. Measles containment is labor-intensive and costly: each case requires investigation and follow-up of many contacts, and quarantines and public information campaigns carry significant costs. A recent analysis of a 2019 measles outbreak in Washington State estimated an average cost of about $47,000 per measles case (including public health response and productivity losses) [35]. Using that figure, more than 1,000 cases in 2025 imposed tens of millions of dollars in societal costs. Most of this expense fell on public health agencies that diverted personnel and resources to the outbreak, as well as on hospitals that treated severe cases. These costs far exceed the investments that would have been needed to maintain high vaccination coverage in the first place, a stark economic argument for prevention [36].

Vulnerable populations and health disparities

Certain groups suffered disproportionately during the measles resurgence. Infants too young to be vaccinated (under 12 months) were at extreme risk; many of the 2025 cases occurred in babies less than a year old, including one of the fatal cases [10]. Children under five years of age in general are highly vulnerable to measles complications like pneumonia and encephalitis; historically, this age group accounts for the majority of measles deaths worldwide [37]. In Samoa’s 2019 outbreak, 87% of measles fatalities were in children under five. The 2025 U.S. outbreak reaffirmed this pattern, as young children made up a large share of hospitalizations and the two pediatric deaths.

Beyond age, immunocompromised individuals are another vulnerable population. People who cannot receive live vaccines due to conditions such as leukemia or immunosuppressive therapy rely on herd immunity for protection [38]. The death of an adult cancer patient in the 2025 outbreak (who contracted measles from his community) underscores that even a single measles case in the wrong setting can be devastating. Pregnant women are also at elevated risk of severe measles and adverse pregnancy outcomes, although fortunately, no pregnant patients died in 2025 [39].

Measles outbreaks tend to exploit social and economic disparities. Underserved communities with limited healthcare access may have lower vaccination rates not out of intent, but because of practical barriers. Parent interviews in the CDC’s National Immunization Survey identified lack of transportation, inconvenient clinic hours, and childcare costs as common obstacles to keeping vaccine appointments [40]. At the same time, several of the worst recent outbreaks (in the U.S. and abroad) have occurred in relatively insular communities (defined by religious or cultural ties) that resist vaccination, regardless of socioeconomic status. Vulnerability to measles is shaped not only by socioeconomic access but also by beliefs and information: California data show personal-belief exemptions cluster in affluent, highly educated communities [6,41], while the 2017 Somali-American outbreak in Minnesota stemmed from autism-related vaccine fears despite free clinic availability. In 2025, some rural counties with healthcare provider shortages experienced delayed diagnoses, which allowed wider spread [42]. Language and cultural barriers further hampered warning efforts in certain immigrant communities. Addressing these issues requires culturally competent engagement well before outbreaks occur. Ultimately, the presence of measles anywhere reveals which populations have been left behind, whether due to marginalization or misinformation, and those gaps must be closed to protect everyone.

The persistence of “zero-dose” children, those who have not received even a first dose of any routine vaccine, has become a key global indicator of immunization system fragility [43]. The undervaccinated pockets that fueled the 2025 U.S. outbreak function much like zero-dose clusters in low- and middle-income countries, suggesting that domestic dashboards could adopt the same metric to flag communities where measles is most likely to resurge.

Lessons learned and policy implications

The 2025 experience has crystallized several lessons for policymakers and public health practitioners. Foremost, maintaining high vaccination coverage is essential. The reemergence of measles in the U.S. vividly demonstrated that even small declines in community immunity can have far-reaching consequences. Therefore, proactive measures to counter vaccine hesitancy should be a permanent fixture of public health work. Such action includes continuous public education that adapts to evolving misinformation, as well as readily accessible vaccination services.

One policy implication is the reconsideration of vaccine exemption policies. States with lenient non-medical exemption (NME) rules observed a higher concentration of unvaccinated children [44], and this outbreak disproportionately affected those clusters. Stricter requirements (for example, only permitting medical exemptions, as California and some other states have done) could be expanded to more jurisdictions. At minimum, simplifying the process for families to vaccinate could help raise immunization rates [45]. Schools and daycare centers are critical enforcement points; ensuring they have the mandate and resources to verify student vaccinations and exclude unvaccinated individuals during outbreaks is key.

Another lesson is the importance of engaging communities and building trust long before health emergencies arise. Combatting misinformation isn’t just about debunking myths in the media; it requires on-the-ground relationships. Local health departments might establish dedicated outreach teams to work with communities known to have lower vaccination rates, partnering with local leaders, clergy, and healthcare providers to deliver consistent pro-vaccine messages [46]. Listening and dialogue are as important as messaging in this effort; people’s concerns must be heard and addressed respectfully to rebuild trust. The outbreak showed that trying to change minds in the midst of a crisis is extremely difficult; the groundwork must be laid in advance.

The outbreak also strengthens the argument for investing in public health infrastructure. The relative containment of measles (it did not become endemic nationwide) was due in part to the rapid mobilization of public health teams. Yet the strain on those teams was apparent: at the height of the response, Texas could field only 2,800 contact tracers, after 400 had been reassigned, against the governor’s target of 4,000 and well below independent estimates of 9,000 to 20,000 needed for statewide control [47]. Policymakers should see these findings as evidence that funding stable epidemiology and immunization programs (not just emergency surge funding) is an investment that pays off. Modernizing immunization information systems to better identify pockets of under-vaccination can enable targeted interventions in the future. For example, predictive modeling studies suggest that without improvements, the U.S. could see measles regaining a foothold within a few decades. One recent model estimated that a sustained 10% decline in MMR coverage could lead to over 11 million measles cases in the U.S. over 25 years [48]. Such scenarios underscore the urgency of taking preventive actions now.

Finally, there is a global dimension to these lessons. Measles anywhere is a threat everywhere. The U.S. will remain at risk of importation as long as measles circulates globally, so supporting international vaccination efforts (through the WHO, the United Nations International Children's Emergency Fund (UNICEF), and other initiatives) aligns with national self-interest [37]. The Samoa and UK experiences show that even relatively small populations can generate large outbreaks that reverberate internationally. Thus, recommitting to the global measles elimination agenda is part of the solution; this includes sharing best practices, data, and resources to bolster measles control in every region.

The 2025 crisis taught us that complacency is dangerous. However, it has also presented an opportunity and acted as a catalyst to fortify policies and programs, ensuring that a disease such as measles doesn't surprise us in the future.

Future directions and recommendations

The priority should and must be to translate these lessons into concrete action [49]. This review’s findings support several recommendations. First, immunization policy should be updated to ensure more uniform coverage, for instance, by narrowing exemption allowances and considering requirements for documentation of immunity in settings like colleges or healthcare employment [38,45]. Second, we recommend enhanced investment in vaccine education and outreach, taking advantage of social media and community networks to spread accurate information and counter myths [50]. Programs that train local trusted figures (teachers, religious leaders, etc.) to advocate for vaccination could be expanded [49]. Third, innovative solutions to improve vaccine uptake should be pursued: for example, research into needle-free vaccine delivery (such as microneedle patches for measles) could reduce barriers and fear, and integrating vaccination reminders into electronic health records and pharmacy systems could increase convenience [51].

It is also advisable to incorporate checks of measles vaccination status into routine healthcare and public health activities. For instance, pediatricians should consistently review and catch up on immunizations at every visit, and public health programs might organize periodic “vaccination days” in communities with low coverage, offering free MMR shots with minimal red tape.

On a national preparedness level, establishing rapid response teams specifically for VPD outbreaks could improve reaction time. These teams could be pre-trained and deployed when clusters of measles (or similar diseases) are detected, much like disaster response units.

Implementation should be audited with clear metrics. Nationally, the CDC already tracks two-dose MMR coverage through SchoolVaxView; the Healthy People 2030 benchmark is ≥ 95% in every state [3,52]. Jurisdictions could add the “7-1-7” timeliness target, detect a cluster within seven days, begin investigation within one day, and complete initial control within seven days as a rapid-response scorecard for VPD outbreaks [53]. Health departments could use county-level dashboards that publish real-time kindergarten MMR and exemption percentages from immunization-information-system (IIS) feeds to trigger outreach whenever coverage drops below the 92% herd-immunity threshold.

Policy experience shows that narrowing NMEs quickly boosts coverage. After California abolished personal-belief exemptions, kindergarten MMR coverage increased from 92.8% to 95.1% in two years [45], while Maine’s 2019 repeal of NMEs produced a 4-point gain, with coverage reaching 97% by 2024-2025 [54].

Internationally, the U.S. can lead by example by re-achieving measles elimination and maintaining it [49]. This means stopping current outbreaks and building a resilient system that quickly addresses any future importation. It also means continuing to support global measles control, such as contributing to global vaccine stockpiles and outbreak response funds and assisting other countries in strengthening their immunization programs.

In the end, the direction is clear: prevent, prevent, prevent. The tools to avoid measles outbreaks exist, and the 2025 crisis has reinforced that we must use them proactively. By doing so, we protect not only our communities but also contribute to the global fight against a virus that no longer needs to cause suffering in the 21st century [55].

## Conclusions

The 2025 U.S. measles outbreak is a sobering reminder that public health victories are never permanent. When population immunity slips, even slightly, vaccine-preventable diseases regain a foothold. Yet the episode also proved that rapid, well-coordinated public health action can still break chains of transmission before measles becomes endemic again. The task ahead is to convert the lessons learned into stronger policies, sustained outreach, and renewed trust in vaccination so we do not repeat this cycle.

Parents’ concerns do not end with disproven autism myths; many remain uneasy about the common, generally mild side effects that follow routine immunization. Addressing those concerns openly and consistently, making side effect profiles part of every informed-consent conversation, has been shown to build confidence rather than erode it. In the future, new methods for administering vaccines, such as microneedle patches or inhaled powders that don't require refrigeration, could make it easier to get vaccinated without needles and help reach people in remote areas. If these methods prove effective, they could help reduce fear and increase vaccination rates. Measles is fully preventable, and the tools to prevent it already exist. It is our collective responsibility to ensure the effective and equitable use of these tools. By doing so, we can turn the setback of 2025 into renewed momentum toward durable measles elimination, protecting today’s children and generations yet to come.
